# Wealth gradient-based divergence in the prevalence of underweight among women by marital status in Quoc Oai district, Vietnam

**DOI:** 10.1080/16549716.2018.1449430

**Published:** 2018-03-28

**Authors:** Jongho Heo, Soo-Young Yu, Jinseon Yi, You-Seon Nam, Dinh Thai Son, Juhwan Oh, Jong-Koo Lee

**Affiliations:** a JW LEE Center for Global Medicine, Seoul National University College of Medicine, Seoul, Republic of Korea; b College of Nursing, Seoul National University, Seoul, Republic of Korea; c Institute for Preventive Medicine and Public Health, Hanoi Medical University, Hanoi, Vietnam

**Keywords:** Socioeconomic, BMI, interaction, LMICs, Hanoi

## Abstract

**Background**: The prevalence of underweight is high among women in Asian countries, despite nutritional changes in the region. Previous studies have demonstrated independent associations between female body weight, marital status and economic status. However, few studies have investigated possible interaction between marital and economic status in relation to Asian women’s body weight.

**Objective**: This study aimed to test associations between household wealth, marital status and underweight among women living in the Quoc Oai district of Vietnam and to identify wealth–marital status interaction in relation to body weight in these women.

**Methods**: Data from 1087 women aged 19–60 years were collected via a baseline community survey conducted in the Quoc Oai district of Hanoi, Vietnam, in 2016. Underweight was defined using an Asian-specific body mass index cut-off (<18.5 kg/m^2^). Marital status was dichotomized into ‘never married’ and ‘ever married.’ Economic status was measured using household wealth index quintiles. Multivariable logistic regressions tested association between wealth and underweight after adjusting for marital status and other confounders. An interaction term (wealth index*marital status) was fitted to determine whether the association between wealth and body weight is modified by marital status.

**Results**: Our results show that underweight was independently associated with a wealth status (odds ratio [OR]: 0.88, 95% confidence interval [CI]: 0.79–0.98, *p *= 0.026) and ever-married status (OR: 0.50, 95% CI: 0.34–0.75, *p *= 0.002). A significant interaction effect (OR: 0.67, 95% CI: 0.50–0.90, *p *= 0.010) indicated that wealthy married women were less likely to be underweight, whereas wealthy never-married women were more likely to be underweight.

**Conclusions**: Our results suggest that the interaction between wealth and marital status has divergent effects on underweight among Asian women. Interventions to reduce underweight among Asian women should simultaneously consider economic and marital status.

## Background

Urbanization, industrialization and socioeconomic growth have led to diet and lifestyle changes in many Asian countries. These changes have led to increases in population body weight and, accordingly, a decrease in the prevalence of underweight. However, some Asian countries are experiencing a double burden of underweight and overweight because disadvantaged groups have been excluded from this nutritional transition [1–3]. The prevalence of underweight remains high (20–40%) among Asian women, despite steady declines over the past decade [4]. Underweight should be managed in Asian women, as low body weight during childbearing years has been associated with health problems affecting both infants and mothers, including preterm birth [5], low birth weight [6,7] and retarded growth during childhood [8]. Additionally this can lead to poor psychological health [9], increased risks of chronic diseases in later life [10] and early mortality [11].

The link between a low economic status and the prevalence of underweight among women in low- or middle-income countries has been well established [4]. Low economic status is associated with insufficient intake of essential nutrients, including protein and fats, which can result in underweight among women of reproductive age [12]. Previous studies in Asian countries demonstrated that the poorest women were approximately two to three times more likely to be underweight than the wealthiest women [13–15]. Moreover, studies have consistently reported clear gradients (stepwise associations) in the prevalence of underweight according to economic status [16–18].

Marital status also has been recognized as an important predictor of a woman’s body weight. Studies in Western countries have demonstrated that among women, unmarried status is associated with a lower body mass index (BMI) [19–21]. Other studies have suggested that women control their weights to maintain a thinner, more attractive physique before marriage [22,23], but tend to gain weight after marriage because of an increase in social interactions and their roles as wives and mothers [24]. However, only a few studies have investigated the relationship between marital status and body weight in Asian countries. Moreover, no study has explored whether an economic–marital status interaction is a possible contributor to Asian women’s body weight.

Vietnam has experienced one of its largest and most rapid economic growth periods during the past 30 years [25]. Improved standards of living have led to increased food expenditures and a lower prevalence of underweight. However, approximately 21.9% of Vietnamese women are underweight [26], particularly in poor rural areas where the prevalence of underweight has been shown to be 23.9% compared with 10.7% in urban areas [26]. Therefore, this study had the following aims: (1) to test the associations of wealth, marital status and underweight among women living in the Quoc Oai district of Hanoi, Vietnam, and (2) to identify the effect of the interaction between wealth and marital status on these associations in these women.

## Methods

### Data source

Data were collected via a baseline community survey conducted in 2016 in the Quoc Oai district of Hanoi, Vietnam as part of the Health System Strengthening Project, which was implemented and led by a collaborative team at Hanoi Medical University (HMU), Ho Chi Minh University of Medicine and Pharmacy of Vietnam (UMP), and Seoul National University (SNU). The Health System Strengthening Project is a five-year intervention project conducted in the north and south of Vietnam. The HMU–UMP–SNU team selected the Quoc Oai district of Hanoi as the project site in the north after consulting with the Hanoi Health Bureau. The Quoc Oai, a rural district is located near the red river region of Hanoi in northern Vietnam and comprises 21 administrative units, including one town and 20 communes, with a total population >180,000 residents (2015).

The community survey was conducted to: (1) describe general socioeconomic and environmental conditions in the district; (2) describe general health status, key health problems, health behaviors and health-related quality of life among the local population; (3) describe health knowledge, attitudes, practices, health care coverage and utilization, and expenditures in the local population; and (4) analyze the association between health status and social determinants of health in the study setting. Household- and individual-level questionnaires were developed by the collaborative research team, and preliminary tests were conducted before undertaking the community survey.

The survey adopted a stratified multistage cluster sampling technique. Probability proportional to size (PPS) sampling was used when selecting clusters (villages) to ensure that the number of selected clusters was proportional to the total population in the district. PPS sampling is useful for ensuring the same probability of inclusion in the sample when the sampling units vary considerably in size [27]. For a given cluster, households were chosen via random selection. In total, 2400 households were randomly selected from 30 clusters in the district. From each household, one person aged 19–60 years was randomly selected; if more than one elderly person (>60 years) lived in a household, an additional person was randomly selected from among the elderly. Data were collected between November 2015 and May 2016. Face-to-face interviews were conducted by trained interviewers under the supervision of staff from the HMU, district health center, or district hospital. Data collected from 2970 individuals from 2353 households were entered into a computer using EpiData 31 (The EpiData Association, Odense, Denmark).

We constructed a dataset of 2640 individuals after merging the household and individual data. After excluding subjects with missing data regarding marital status (n = 4) and occupation (*n* = 20), 2616 individuals were enrolled in the study (1197 men and 1419 women). We subsequently excluded men to focus on the women’s weight status. Additionally, we excluded women older than 60 years (*n* = 332) because of possible reporting bias [27,28]. Table 1 presents descriptive statistics for the final study sample of 1087 women aged between 19 and 60 years.

**Table 1.** Characteristics of the study population of women aged 19–60 years who participated in the Quic Oai Community Survey in 2016 (*n* = 1087).

		*n*	%
Underweight (<18.5 kg/m^2^)		154	14.2
Marital status	Never married	221	20.3
	Ever married	866	79.7
Wealth index quintile	1	211	19.4
	2	207	19.0
	3	203	18.7
	4	224	20.6
	5	242	22.3
Age (years)	19–30	298	27.4
	31–40	307	28.2
	41–50	205	18.9
	51–60	277	25.5
Race/ethnicity	Kinh	805	74.1
	Other	282	25.9
Education	≤Primary	264	24.3
	Secondary	421	38.7
	≥High school graduation	402	37.0
Job status	Manual	975	89.7
	White collar	23	2.1
	Unemployed or other	89	8.2
Household size	1–2	144	13.3
	3–4	473	43.7
	5–6	358	33.1
	≥7	1.8	9.9

### Measurement

#### Underweight

Underweight was defined using an Asian-specific BMI cut-off value of <18.5 kg/m^2^ [29,30]. The BMI was calculated as the self-reported body weight in kilograms divided by the self-reported height in meters squared [30].

#### Wealth index

The (household) wealth index is widely used to measure the socioeconomic status of individuals in developing countries where income and expenditure data are often not available [31]. This index is a composite measure generated through a principal-components analysis of key aspects of living standards, including the number of household members, ownership of material goods, housing quality, water and sanitation quality, and access to energy sources [31]. A high wealth index indicates better economic status. We used wealth index quintiles in our models (range: 1–5).

#### Marital status

We dichotomized marital status into never married (reference) and ever married by collapsing the categories of married, separated, divorced and widowed.

#### Other covariates

We used age, race/ethnicity, education level, employment status and household size as covariates. Age was grouped into four categories: 19–30 years (reference), 31–40 years, 41–50 years and 51–60 years. Race/ethnicity was dichotomized into Kinh (reference) or others (Muong, Thai and Tay). Education level was grouped into to three categories: less than primary level (reference), secondary and more than high school graduation. The respondents’ employment status was categorized as manual (reference), white collar and unemployed or other (student, retired and other). Household size was categorized into four groups: 1–2, 3–4, 5–6 and ≥7 people.

#### Statistical analysis

First, the prevalence of underweight was calculated using the above-mentioned BMI cut-off for the Asian population. Subsequently, multivariate logistic regression was conducted to identify wealth gradients among underweight participants, using Stata’s svy commands to adjust the standard errors for the intragroup correlation (Model 1). Second, an interaction term, wealth index*marital status was added to the model, which was run again to test whether wealth gradients varied significantly among underweight participants according to marital status (Model 2). After assessing the statistical significance of the interaction term, the predicted probabilities and confidence intervals for underweight were estimated and graphed for each level of the wealth index marital status using bootstrap values and the percentile method with 1000 random sample replications [32]. The analyses were performed using Stata version 14.2 (Stata Corp, College Station, TX, USA).

## Results

Table 1 presents the characteristics of the respondents to the community survey (*n* = 1087 women). The prevalence of underweight among the female respondents was 14.2%. Approximately 80% of women were ever married. The major racial/ethnic group was Kinh (74.1%), and more than 75% of women had an education level higher than primary school graduation.

Table 2 presents the results of the multivariate logistic regression models. In Model 1, a one-level increase in the wealth index quintile was associated with a 12% lower likelihood of underweight [OR: 0.88, 95% confidence interval (CI): 0.79–0.98, *p *= 0.026]. Being ever married was also associated with a lower risk of underweight (OR: 0.50, 95% CI: 0.34–0.75, *p *= 0.002), compared with being never married. Kinh ethnicity was associated with a higher risk of underweight, compared with other racial/ethnic groups (OR: 5.25, 95% CI: 3.20–8.60, *p *< 0.001). In Model 2, the interaction effect of the wealth index and marital status on underweight was significant (OR: 0.67, 95% CI: 0.50–0.90, *p *= 0.010).

**Table 2.** Multivariable logistic regression of factors associated with underweight in women aged 19–60 years who participated in the Quic Oai Community Survey in 2016 (*n* = 1087).

	Model 1			Model 2		
	OR	95% CI	*p*	OR	95% CI	*p*
Wealth index	0.88	0.79 0.98	0.026	1.18	0.95 1.48	0.124
Wealth index*marital status				0.67	0.50 0.90	0.010
Age (ref. 19–30 years)						
31–40	0.50	0.33 0.77	0.004	0.54	0.35 0.83	0.008
41–50	0.48	0.29 0.82	0.010	0.49	0.28 0.84	0.013
51–60	0.84	0.46 1.55	0.555	0.84	0.46 1.54	0.552
Race/ethnicity (ref. other)						
Kinh	5.25	3.20 8.60	<0.001	5.36	3.20 8.97	<0.001
Marital status (ref. never married)						
Ever married	0.50	0.34 0.75	0.002	1.62	0.67 3.91	0.261
Education (ref. ≤primary)						
Secondary	0.92	0.57 1.49	0.732	0.91	0.57 1.48	0.696
≥High school graduation	1.07	0.69 1.64	0.757	1.06	0.70 1.60	0.782
Job status (ref. manual)						
White collar	0.93	0.24 3.53	0.907	0.87	0.20 3.80	0.847
Unemployed or other	1.15	0.56 2.36	0.680	1.06	0.50 2.24	0.871
Household size (ref. 1–2)						
3–4	1.08	0.69 1.68	0.731	0.93	0.57 1.52	0.772
5–6	1.03	0.63 1.68	0.888	0.91	0.57 1.46	0.679
≥7	1.12	0.50 2.50	0.771	0.93	0.37 2.37	0.876

Model 1: adjusted variables include age, race/ethnicity, marital status, education, job status and household size. Model 2: adjusted variables include those in model 1, as well as the interaction variable between the wealth index and marital status.

OR: odds ratio; CI: confidence interval.

**Figure 1.**
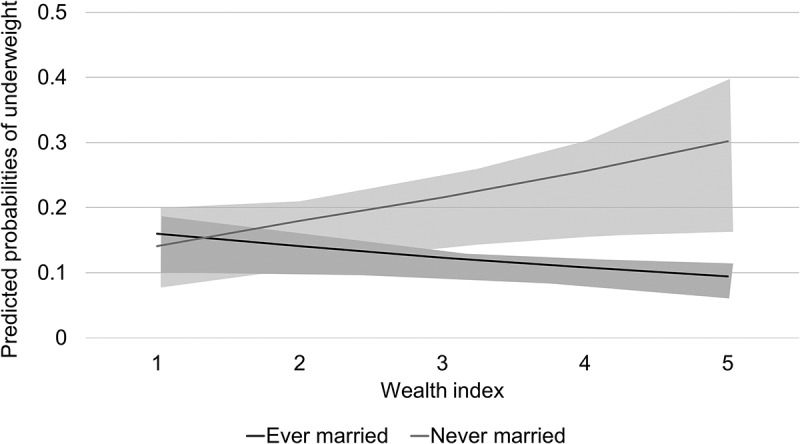
Predicted probabilities and 95% confidence intervals (shading) of underweight across wealth index quintiles and according to marital status among women aged 19–60 years who participated in a community survey in Quoc Oai, Vietnam in 2016.

Figure 1, which presents an interpretation of these interaction effects, demonstrates variations in the predicted probabilities of being underweight in accordance with both wealth gradients and marital status. A positive relationship was observed between the wealth index and underweight among the never-married participants. In contrast, a negative relationship was observed between the wealth index and underweight among women who were ever married.

## Discussion

This study examined associations between household wealth, marital status and underweight in women living in the Quoc Oai district of Vietnam and further assessed the effect of the interaction between wealth and marital status to determine whether wealth gradients diverged among ever- and never-married women. Overall, we observed a negative relationship between the wealth index and underweight among female survey respondents. However, when we stratified the sample by marital status (ever married or never married), women who were never married were more likely to remain underweight as the wealth index increased, whereas those who were ever married were less likely to be underweight as the wealth index increased.

These patterns are consistent with previous studies from Western countries, where the ‘never married’ status and high income were associated with a low BMI among women [33,34]. In Westernized countries, where thin women are often considered more attractive, unmarried women may use their economic resources to maintain a lean physique by living in neighborhoods that promote physical activity and purchasing health-promoting goods and healthy foods [35]. Several studies of Vietnamese youth reported that they preferred a slim body image, in contrast to older generations. A study of body image among Vietnamese female adolescents revealed that they also preferred a slim physique, despite understanding the health risks [36]. Another study of Vietnamese university students in urban areas demonstrated that underweight female students were more satisfied with their body image, compared with normal weight or overweight students [37]. These results imply that young Vietnamese women who have never been married may use their economic resources to remain thin and thus appear attractive. Alternatively, this pattern may also be explained by women who have married because of high BMI. In traditional Vietnamese culture, a woman’s plump appearance is associated with health, wealth and food security, and has therefore been considered attractive [38].

These patterns may also be attributable to the stigmatization of women who fail to find a husband by a certain age. The Vietnamese word, ‘*echong*’, refers to women older than the marriageable age by Vietnamese standards. The patriarchal culture in Vietnam stigmatizes women for their ‘failure’ to find a husband in a timely manner, as Vietnamese women become ‘real’ adults and gain social recognition upon becoming wives and mothers [39]. Stereotypes such as a poor temper, selfishness, fussiness in choosing a spouse, unwillingness to fulfill their womanly functions in households and even perhaps sexual ‘abnormality’ may negatively affect a woman’s physical and/or psychological health, resulting in underweight. This stigmatization and neglect may be intensified in wealthy families, which may experience societal shame because of the unmarried women. Alternatively, women who no longer seek marriage may not maintain a normal weight because doing so is expensive and/or because they prefer to save money [19].

In addition, our study demonstrated a negative relationship between wealth and underweight among the ever-married group. This finding is consistent with previous studies from the United States in which married women are more likely to have a high BMI [20,24]. Previous studies from the United States have suggested that marriage protects a woman’s health by providing greater economic resources and social support, which can facilitate access to healthy foods or the adoption of healthy behaviors [40]. In Vietnam, married women are expected to have children without intentional birth delays. To ensure a successful pregnancy, married women of fertile age may eat well and avoid risky behaviors such as heavy domestic work, and these practices may enable wealthy women to have successful pregnancies. Moreover, the patriarchal culture in Vietnam may evoke married women’s social obligations as wives and mothers, and thus encourage them to prepare healthy foods for their families and eat regular meals. These obligations may also be held by widowed, separated and divorced women. In addition, wealthy women may be less likely to be underweight because of the increased availability of resources and environments that support these social obligations.

Our study also found that the Kinh ethnic group was more likely to be underweight after controlling for other covariables. By contrast, a previous descriptive study reported that the prevalence of underweight was significantly higher among women in minority ethnicities than among the Kinh ethnic group [41]. Although the reason for this discrepancy is unclear, this counterintuitive finding may be explained by food intakes of minority ethnic groups during festivals, which anthropological studies of minority ethnicities in Vietnam have identified as an important part of ethnic minority cultures and living standards [42,43]. Another study that explored the financial expenditures of minority ethnic groups using the Vietnam Living Standards Survey revealed that ethnic minorities spent 13% more on food during festivals and other holidays, compared with the Kinh ethnic group [44]. Further studies are needed to better understand ethnic differences with respect to women’s body weight. Other covariables, including education, job status and family size, were not significantly associated with underweight.

This study had several limitations. First, given the cross-sectional data structure, causation cannot be established regarding association between underweight, wealth and marital status. Second, our findings cannot be generalized to women in other regions of Vietnam, especially southern Vietnam, which differs from the northern part in terms of the culture, climate and degree of Westernization [45]. Third, as all variables used in this study were self-reported without additional validation, the data should be cautiously interpreted because of possible bias from socially desirable responses and selective memory. A follow-up survey with body measurements may be needed to validate the participants’ self-reported heights and weights. Future longitudinal studies should also investigate whether women who were previously underweight had gained weight once they were married. Despite these limitations, however, our study contributes to the literature regarding the relationship between socioeconomic gradients and underweight among Asian women. There is a need to investigate these associations further by conducting similar studies in other Asian countries. To the best of our knowledge, this is the first study of its kind to provide evidence of a wealth–marital status interaction effect on the body weight of Asian women.

## Conclusions

In conclusion, this study found that both low economic status and being never married were independently associated with underweight in a sample of Vietnamese women, although wealth and marital status interact. These findings are important for improving our understanding of the dynamics of underweight and for tailoring targeted underweight prevention strategies for women in the Vietnamese population. Interventions or policies to address underweight among women have generally assumed a negatively graded association between economic status and underweight. However, our study suggests that the moderating effect of a woman’s marital status should be considered.

## Paper context

Although existing studies have shown association between marital and economic status with body weight among women, few studies have examined a possible wealth–marital status interaction when addressing underweight in low- and middle-income countries. This study of Vietnamese women found that the combination of being wealthy and ever married was inversely associated with underweight and that being wealthy and never married was positively associated with underweight. Efforts to reduce socioeconomic inequality and its effects on underweight in women in Vietnam should also consider marital status.
